# CLIMBER: Climatic niche characteristics of the butterflies in Europe

**DOI:** 10.3897/zookeys.367.6185

**Published:** 2014-01-06

**Authors:** Oliver Schweiger, Alexander Harpke, Martin Wiemers, Josef Settele

**Affiliations:** 1 Helmholtz Centre for Environmental Research – UFZ, Department of Community Ecology, Theodor-Lieser-Strasse 4, 06120 Halle, Germany; 2 iDiv, German Centre for Integrative Biodiversity Research (iDiv) Halle-Jena-Leipzig, Deutscher Platz 5e, 04103 Leipzig, Germany

**Keywords:** Climate change, climate warming, CTI, global change, global warming, modelling, risk, trend, STI, Europe, butterflies, Lepidoptera, Papilionidae, Pieridae, Lycaenidae, Riodinidae, Nymphalidae, Hesperiidae

## Abstract

Detailed information on species’ ecological niche characteristics that can be related to declines and extinctions is indispensable for a better understanding of the relationship between the occurrence and performance of wild species and their environment and, moreover, for an improved assessment of the impacts of global change. Knowledge on species characteristics such as habitat requirements is already available in the ecological literature for butterflies, but information about their climatic requirements is still lacking. Here we present a unique dataset on the climatic niche characteristics of 397 European butterflies representing 91% of the European species (see Appendix). These characteristics were obtained by combining detailed information on butterfly distributions in Europe (which also led to the ‘Distribution Atlas of Butterflies in Europe’) and the corresponding climatic conditions. The presented dataset comprises information for the position and breadth of the following climatic niche characteristics: mean annual temperature, range in annual temperature, growing degree days, annual precipitation sum, range in annual precipitation and soil water content. The climatic niche position is indicated by the median and mean value for each climate variable across a species’ range, accompanied by the 95% confidence interval for the mean and the number of grid cells used for calculations. Climatic niche breadth is indicated by the standard deviation and the minimum and maximum values for each climatic variable across a species’ range. Database compilation was based on high quality standards and the data are ready to use for a broad range of applications.

It is already evident that the information provided in this dataset is of great relevance for basic and applied ecology. Based on the species temperature index (STI, i.e. the mean temperature value per species), the community temperature index (CTI, i.e. the average STI value across the species in a community) was recently adopted as an indicator of climate change impact on biodiversity by the pan-European framework supporting the Convention on Biological Diversity (Streamlining European Biodiversity Indicators 2010) and has already been used in several scientific publications. The application potential of this database ranges from theoretical aspects such as assessments of past niche evolution or analyses of trait interdependencies to the very applied aspects of measuring, monitoring and projecting historical, ongoing and potential future responses to climate change using butterflies as an indicator.

## Introduction

Global change seriously threatens biodiversity at all organisational levels ranging from genetic diversity, performance and occurrence of single species, taxonomic, phylogenetic and functional diversity of communities and species assemblages to properties of whole ecosystems including the provision of ecosystem services for human well-being (Lavergne et al. 2010; Parmesan 2006; Potts et al. 2010; Schröter et al. 2005). But species are not equally at risk when facing global change (e.g. Settele et al. 2008). In the context of climate change, several species-specific ecological characteristics have been identified to determine vulnerability, including diets, habitat requirements, ecological specialisation and plasticity and the ecological characteristics of interacting species (Heikkinen et al. 2010; Pöyry et al. 2009; Schweiger et al. 2012; Visser 2008; Warren et al. 2001). Thus, good knowledge of the ecological characteristics relevant for the reaction of species and communities to particular drivers of global change is needed, which can then be utilised as powerful indicators for conservation planning and action.

**Figure 1.**
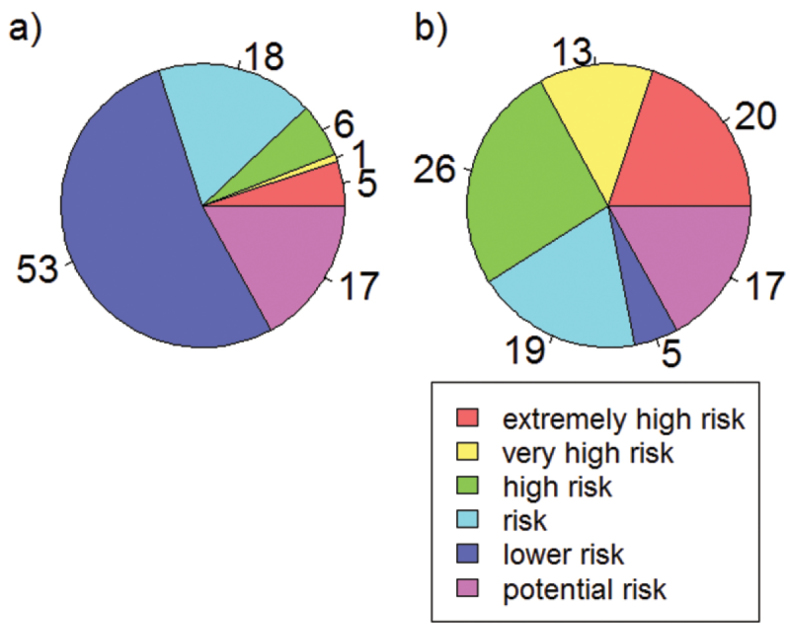
Proportion of species (%) with different climatic risk status after Settele at al. (2008) assuming full dispersal (**a**) and no dispersal capacity (**b**).

One of the most important ecological characteristics to assess how species react to climate change obviously is the climatic niche. While knowledge on particular species characteristics such as habitat requirements is already available for some species groups, crucial publicly available information about climatic requirements is still lacking for the majority of the species. Here we present a unique dataset on climatic niche characteristics of 397 (91%) butterfly species in Europe, which have been shown to be particularly sensitive to changing climates (Hill et al. 2002; Settele et al. 2008; Warren et al. 2001). Based on projections of future suitable climatic conditions, Settele et al. (2008) showed that under the assumption of unlimited dispersal 7% of the European butterflies are at an extremely high or very high risk (i.e. a loss of more than 95% and 85%, respectively of their current range size until 2080), 6% are at high risk (>70% loss) and 18% are at risk (>50% loss; Fig. 1). However, the more realistic assumption of no dispersal (in the given amount of time) projected 33% of the butterflies to be at an extremely high or very high risk, 26% to be at high risk and 19% to be at risk (Fig. 1).

**Figure 2.**
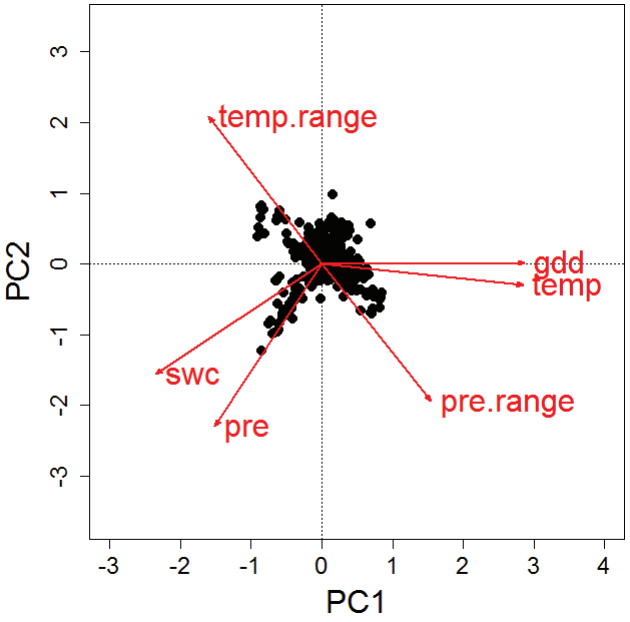
Results from a principal component analysis of the species-specific mean values of six different climate variables. Mean values per species have been calculated based on the observed records per 50 km × 50 km CGRS grid cell across a species’ European distribution. PC1 explained 58% and PC2 32% of the variability. Niche characteristics according to annual temperature (temp) and growing degree days until August (gdd) are highly correlated. Also, the two measures of water availability, annual precipitation (pre) and soil water content (swc) show some similarity, while the indicators of annual range in precipitation (pre.range) and temperature (temp.range) are negatively correlated. In spite of these similarities, aspects of energy, water and their annual variability can be assessed independently with a choice of at least three of the indicators.

Based on detailed data on the distribution of European butterflies, which also led to the ‘Distribution Atlas of European Butterflies’ (Kudrna 2002), the ‘Climatic Risk Atlas of European Butterflies (Settele et al. 2008) and the ‘Distribution Atlas of Butterflies in Europe’ (Kudrna et al. 2011), we extracted measures of climatic conditions (indicating niche breadth and position) within the distributional range of each species. As a consequence of this approach, users of this dataset should be aware that the provided measures refer to the realised climatic niche and not to the fundamental niche (sensu Hutchinson 1957; but see discussion in Araújo and Guisan 2006). The extracted measures reflect two primary properties of climate, energy and water, which are known to affect butterfly species performance and distributions as a consequence of physiological limitations (Buckley et al. 2011; Roy et al. 2001). Most of these measures are quite independent from each other and cover different aspects of the climatic niche (Fig. 2).

By combining a comprehensive database on the distribution of European butterflies with publicly available climatic data in combination with a constantly high level of quality control at crucial steps of the data generation, CLIMBER represents a unique and ready-to-use dataset for a broad variety of potential applications. Analysis of phylogenetic signals in the climatic niche characteristics, for instance, can be used to assess past niche evolution which can lead to projections of potential future risks in the face of rapid climate change (for a comparable analysis for birds see Lavergne et al. 2013). Also, analyses relating climatic niche properties to other species traits can be helpful to assess interdependencies of different ecological characteristics, as has been done recently for birds and their temperature and habitat preferences (Barnagaud et al. 2012). So far the most powerful application of climatic niche characteristics provided in this dataset comes from the ‘species temperature index’ (STI). The STI is simply the mean temperature value per species across its range. Based on the STI, the ‘community temperature index’ (CTI) has been suggested as a powerful and robust tool to measure the response of local communities to temperature change (Devictor et al. 2008; Devictor et al. 2012a; Devictor et al. 2012b). The CTI is calculated as the average STI value across the species or specimens in a community and has been used to analyse the temporal response to climate warming of local bird and butterfly communities across Europe. One striking result of this study was the detection of time lag effects in the community response to climate warming and that these lag effects differed between the two species groups (Devictor et al. 2012a).

STI values for European butterflies can be of great value for governmental and non-governmental conservation organisations (Van Swaay et al. 2010; Van Swaay et al. 2008). Based on the STI, the CTI was recently adopted as an indicator of climate change impact on biodiversity by the pan-European framework supporting the Convention on Biological Diversity (Streamlining European Biodiversity Indicators 2010; http://ec.europa.eu/environment/nature/knowledge/eu2010_indicators). Thus, STI and corresponding CTI values can perfectly complement and enrich the analysis of all kind of butterfly monitoring schemes. To address the fact that temperature is not the only changing climatic factor or aspect of the climatic niche, we think that the additionally provided climatic niche characteristics concerning water availability and annual climatic variability can help to enrich the landscape of target-specific analyses and indicators (Fig. 2). By providing public access to this dataset, we hope to contribute to improvements of the scientific understanding of how climate change affects species and communities and to improve monitoring and conservation actions for climate change mitigation.

## Metadata

For the description of the metadata we followed the standards suggested by Michener et al. (1997) in a slightly modified way.

## Title

CLIMBER: Climatic niche characteristics of the butterflies in Europe

## Contributors

### Dataset owner

Oliver Schweiger, Alexander Harpke, Martin Wiemers, Josef Settele

Helmholtz Centre for Environmental Research – UFZ, Department of Community Ecology, Theodor-Lieser-Strasse 4, 06120 Halle, Germany

### Contact person

Oliver Schweiger

Affiliation: Helmholtz Centre for Environmental Research – UFZ, Department of Community Ecology

Address: Theodor-Lieser-Strasse 4, 06120 Halle, Germany

Phone: +49 345 558 5306

Email: oliver.schweiger@ufz.de

## Geographic, temporal and taxonomic coverage

### Geographic coverage and spatial resolution

**Figure 3.**
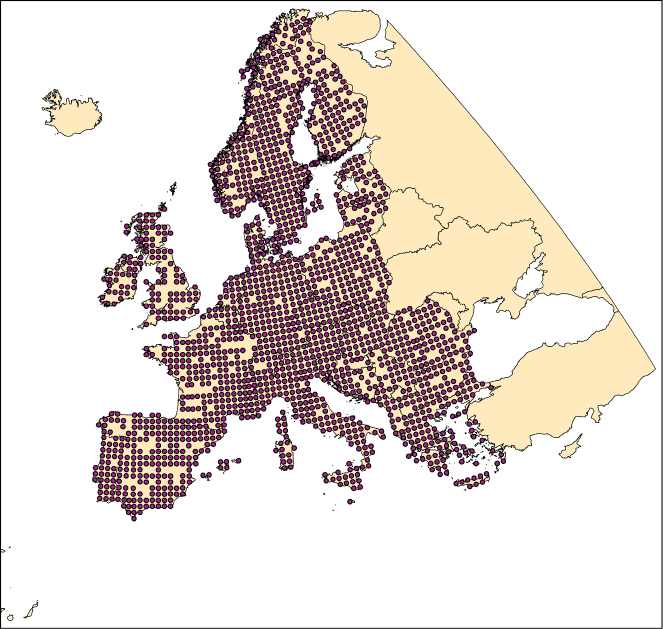
Geographic coverage used for the calculation of the climatic species characteristics. Purple dots indicate 50 km × 50 km CGRS grid cells with available species records.

Climatic niche characteristics are provided for all butterfly species occurring within a European window of 11°W, 32°E longitude and 34°N, 72°N latitude (Fig. 3). Resolution of butterfly distribution and corresponding climate data used to calculate climatic niche characteristics corresponds to the 50 km × 50 km Common European Chorological Grid Reference System (CGRS; http://www.eea.europa.eu/data-and-maps/data/common-european-chorological-grid-reference-system-cgrs).

The geographic window excludes data from the Atlantic islands under European administration (the Azores, Madeira and Canary Islands) as well as Cyprus and Iceland. Due to low levels of recording, data from Belarus, Ukraine, Moldova, and Russia were also excluded. Additionally, no climate data were available for two species with extremely local distributions on the Pontine Islands and the Greek island of Nissiros. These restrictions led to the exclusion of 38 of the European butterfly species listed in Kudrna et al. (2011), but confined to these regions (Table 1).

**Table 1.** Species occurring in Europe and listed in Kudrna et al. (2011) but not considered for the assignment of climatic niche characteristics in this database.

Species	European range
*Azanus ubaldus* (Stoll, 1782)	Canary Islands
*Catopsilia florella* (Fabricius, 1775)	Canary Islands
*Chazara persephone* (Hübner, [1805])	Ukraine
*Chilades galba* (Lederer, 1855)	Cyprus
*Cigaritis acamas* (Klug, 1834)	Cyprus
*Cyclyrius webbianus* (Brulle, 1839)	Canary Islands
*Euchloe eversi* Stamm, 1963	Canary Islands
*Euchloe grancanariensis* Acosta, 2008	Canary Islands
*Euchloe hesperidum* Rothschild, 1913	Canary Islands
*Glaucopsyche paphos* Chapman, 1920	Cyprus
*Gonepteryx cleobule* (Hübner, 1825)	Canary Islands
*Gonepteryx eversi* Rehnelt, 1974	Canary Islands
*Gonepteryx maderensis* Felder, 1863	Madeira
*Gonepteryx palmae* Stamm, 1963	Canary Islands
*Hipparchia azorina* (Strecker, 1899)	Azores
*Hipparchia bacchus* Higgins, 1967	Canary Islands
*Hipparchia cypriensis* (Holik, 1949)	Cyprus
*Hipparchia gomera* Higgins, 1967	Canary Islands
*Hipparchia maderensis* (Bethune-Baker, 1891)	Madeira
*Hipparchia sbordonii* Kudrna, 1984	Pontine Islands
*Hipparchia tamadabae* Owen & Smith, 1992	Canary Islands
*Hipparchia tilosi* (Manil, 1984)	Canary Islands
*Hipparchia wyssii* (Christ, 1889)	Canary Islands
*Hypolimnas misippus* (Linnaeus, 1764)	Canary Islands
*Maniola cypricola* (Graves, 1928)	Cyprus
*Maniola halicarnassus* Thomas, 1990	Nissiros Island
*Neolycaena rhymnus* (Eversmann, 1832)	Ukraine
*Pararge xiphia* (Fabricius, 1775)	Madeira
*Pararge xiphioides* Staudinger, 1871	Canary Islands
*Pieris cheiranthi* (Hübner, 1808)	Canary Islands
*Pieris wollastoni* Butler, 1866	Madeira
*Polyommatus corydonius* (Herrich-Schäffer, 1852)	Ukraine
*Polyommatus damocles* (Herrich-Schäffer, 1844)	Ukraine
*Polyommatus damone* (Eversmann, 1841)	Ukraine
*Pseudochazara euxina* (Kusnezov, 1909)	Ukraine
*Thymelicus christi* Rebel, 1894	Canary Islands
*Tomares callimachus* (Eversmann, 1848)	Ukraine
*Vanessa vulcania* (Godart, 1819)	Canary Islands & Madeira

### Temporal reference period

**Figure 4.**
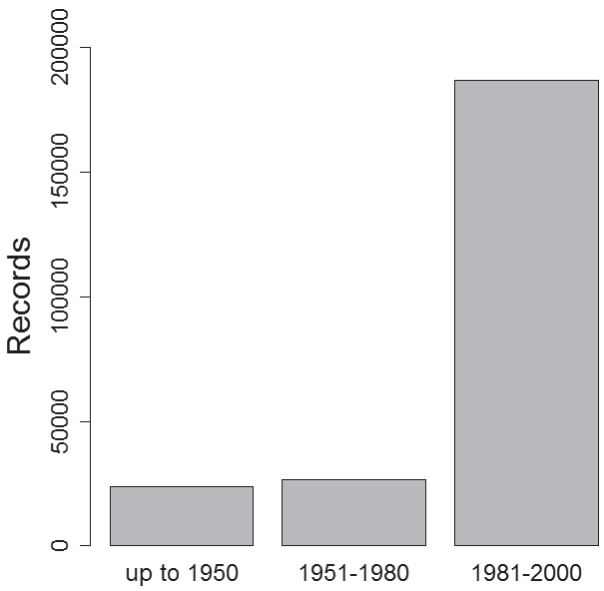
Temporal availability of records and corresponding sampling intensity. Only the period of 1981–2000 has been considered in CLIMBER.

Only butterfly distribution data from the period of 1981 to 2000 were considered due to low sampling intensity in earlier periods (Fig. 4) and to minimize errors due to ongoing range shifts as a response to recent climate change.

## Taxonomy

### Taxonomic ranks

**Phylum:** Arthropoda

**Subphylum:** Hexapoda

**Class:** Insecta

**Order:** Lepidoptera

**Superfamily:** Papilionoidea (sensu Regier et al. 2013; Wahlberg et al. 2013)

**Families:** Hesperiidae, Lycaenidae, Nymphalidae, Papilionidae, Pieridae, Riodinidae

**Common name:** butterflies

## Taxonomic coverage

**Figure 5.**
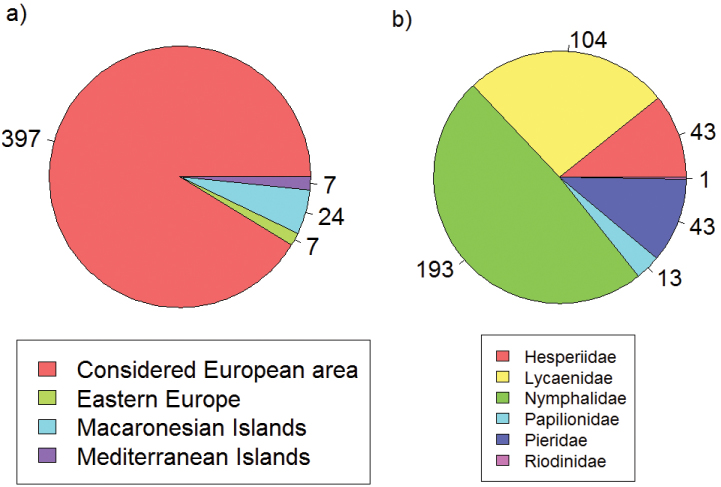
Taxonomic coverage according to the entire European butterfly fauna (**a**) and families (**b**). Values indicate number of species.

The taxonomic coverage spans all butterfly species within the selected geographic window (397 species) and represents 91% of all European species (Fig. 5). Thirty-eight species from less well sampled Eastern European countries, Atlantic and small Mediterranean islands have not been considered (Fig. 5a). The taxonomy of European butterfly species follows Kudrna et al. (2011). Erroneous use of brackets around authors’ names was corrected in 15 cases (cf. Tshikolovets 2011; Table 2).

**Table 2.** Corrected species names (cf. Tshikolovets 2011) in comparison toKudrna et al. (2011).

Corrected species names
*Anthocharis damone* Boisduval, 1836
*Apatura metis* Freyer, 1829
*Argynnis elisa* Godart, 1823
*Aricia morronensis* Ribbe, 1910
*Cacyreus marshalli* Butler, 1898
*Colias aurorina* Herrich-Schäffer, 1850
*Erebia ottomana* Herrich-Schäffer, 1847
*Maniola chia* Thomson, 1987
*Maniola halicarnassus* Thomson, 1990
*Melitaea asteria* Freyer, 1828
*Melitaea varia* Meyer-Dür, 1851
*Pararge xiphioides* Staudinger, 1871
*Plebejus trappi* (Verity, 1927)
*Pseudochazara amymone* Brown, 1976
*Pseudochazara orestes* Prins & Poorten, 1981

*Aricia artaxerxes* (Fabricius, 1793) and *Aricia montensis* Verity, 1928 are treated in CLIMBER as distinct species with parapatric distributions (see Sanudo-Restrepo et al. 2013). The latter species is confined to the Iberian Peninsula and North Africa.

For the local Macedonian endemic *Pseudochazara amymone* Brown, 1976 no data were available for the considered time period. After its first discovery in Greece in 1975, the species was not reliably recorded again until its recent rediscovery in Southern Albania (Eckweiler 2012). According to Eckweiler (2012), *Pseudochazara amymone* should be considered a subspecies of *Pseudochazara mamurra* (Herrich-Schäffer, [1846]), which is widespread in the Middle East.

The following species in our database actually comprise records of more than one species, most of which were recognized only recently, and are difficult or impossible to distinguish without genitalia examination or molecular methods.

- *Carcharodus alceae* (Esper, 1780) probably contains data of the sibling species *Carcharodus tripolinus* (Verity, 1925) from the Southern Iberian Peninsula, differing only in genitalia characters.
- *Leptidea sinapis* (Linnaeus, 1758) is a complex of three sibling species, and includes data of *Leptidea juvernica* Williams, 1946, and *Leptidea reali* Reissinger, 1990 (Dincă et al. 2011b; Dincă et al. 2013). Whereas *Leptidea sinapis* can be separated by their genitalia, the other two taxa can only be separated from each other by molecular characters. *Leptidea reali* seems to replace *Leptidea juvernica* in SW Europe, and both occur largely in sympatry with *Leptidea sinapis*.
- *Lycaena tityrus* (Poda, 1761) includes data of *Lycaena bleusei* Oberthür, 1884 from Central Spain and Central Portugal, which appears to be a distinct species according to unpublished molecular data.
- *Melitaea athalia* (Rottemburg, 1775) includes the Southwest European *Melitaea nevadensis* Oberthür, 1904 (syn. *celadussa* Fruhstorfer, 1910) which might only be a subspecies of the former. Molecular data are inconclusive regarding the taxonomic status of these parapatric taxa.
- *Melitaea phoebe* (Goeze, 1779) recently turned out to be a complex of at least two largely sympatric species with distinctive larval colouration, and our data probably include records of *Melitaea ornata* Christoph, 1893 (syn. *telona* Fruhstorfer, 1908 and *emipunica* Verity, 1919) (see Toth et al. 2013; Toth and Varga 2011; Tshikolovets 2011).
- *Polyommatus icarus* (Rottemburg, 1775) includes data of *Polyommatus celina* (Austaut, 1879), which was recognized as a distinct species from North Africa and the Canary Islands by molecular methods (Wiemers et al. 2010), but also occurs in Southern Spain, and appears to replace *Polyommatus icarus* in the Balearic Islands, Sardinia, and Sicily (Dincă et al. 2011a).
- *Pontia daplidice* (Linnaeus, 1758) includes the data of the sibling species *Pontia edusa* (Fabricius, 1777), a parapatric taxon, which can only be distinguished by molecular methods (Geiger and Scholl 1982; John et al. 2013; Wiemers unpubl.).

## Methods

### Butterfly distribution data

**Figure 6.**
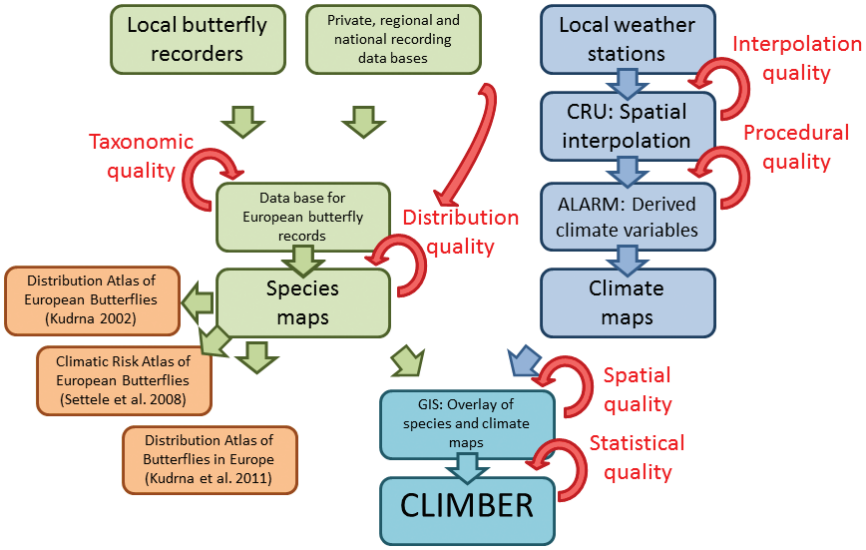
Work flow and data sources for the generation of CLIMBER. Butterfly distribution data are based on a database which combines information from local recorders and private, regional and national databases. Thereof, species distributional maps have been developed. Together with maps of original and derived climate variables, based on interpolated data from local weather stations, species distribution-climate relationships have been assessed in a GIS. Based on these relationships several statistics describing the climatic characteristics of 397 European butterfly species have been developed and stored in CLIMBER. Several steps of quality control ensure a high level of data accuracy. CRU; Climate Research Unit, University of East Anglia (http://www.cru.uea.ac.uk/). ALARM; EU, FP6 project ‘Assessing Large Scale Risks for Biodiversity with Tested Methods’ (http://www.alarmproject.net/climate/climate/).

Climatic niche characteristics of the butterflies in Europe are based on their European distribution. Butterfly distributions were available from about 7000 georeferenced localities and about 200,000 database records. These records were stored in a database and constituted also the basis for ‘The Distribution Atlas of European Butterflies’ (Kudrna 2002) and, as an updated version, for the ‘Distribution Atlas of Butterflies in Europe’ (Kudrna et al. 2011; Fig. 6). The data are owned by the Helmholtz Centre for Environmental Research (and thus by the originators of CLIMBER). To avoid problems of occasional undersampling and imprecise geo-reference of some locations at the local scale, we re-sampled the localities to 1720 CGRS grid cells at a 50 km × 50 km resolution. Distribution data refer to the period of 1981–2000 and cover the abovementioned European window of 11°W, 32°E longitude and 34°N, 72°N latitude. We also provide an estimation of species range sizes by the number of grid cells used for calculating the climatic species characteristics.

### Climate data

We used monthly, interpolated climate data (publicly available at http://www.alarmproject.net/climate/climate), originally provided via the ALARM project (Settele et al. 2012; Settele et al. 2005; Spangenberg et al. 2012) at a 10 arcmin grid resolution (Mitchell et al. 2004; New et al. 2000) and aggregated them to the CGRS grid (Fig. 6). For a detailed description of the climate data see Fronzek et al. (2012). The following basic climatic variables were used to assess aspects of the climatic niche: mean annual temperature (°C), range of annual temperature (°C), annual precipitation sum (mm), range of annual precipitation (mm), accumulated growing degree days with a base temperature of 5°C until February, April, June and August and soil water content for the upper horizon (0.5 m). Different time periods for calculating accumulated growing degree days enable the consideration of different phenologies and phenological aspects in the analysis of the climatic species characteristics. We do not provide growing degree days for periods ending later than August because these values are highly correlated with mean annual temperature in any case. Soil water content originated from the dynamic vegetation model LPJ-GUESS (Hickler et al. 2009; Hickler et al. 2004) which provides a process-based representation of the water balance in terrestrial ecosystems. According to the time period of the butterfly distribution data, we used averaged values for the period 1971–2000 for the climate data.

### Calculation of the climatic niche characteristics

Climatic niche characteristics were calculated per butterfly species according to the climatic conditions across their respective ranges, i.e. across all grid cells in which a particular species occurs (see Devictor et al. 2012a; Schweiger et al. 2012; Van Swaay et al. 2010; Van Swaay et al. 2008; Fig. 6). The dataset comprises information for the position and breadth of the climatic niche. Niche position is indicated by the median and mean value for each climate variable across a species’ range, accompanied by the 95% confidence interval for the mean. Niche breadth is indicated by the standard deviation and the minimum and maximum values for each climatic variable across a species’ range.

### Data verification

Several steps of quality control ensure a high level of data accuracy (Fig. 6). During the step of compiling butterfly records for Europe, taxonomic experts addressed problems of potential misidentification, synonymy and the taxonomic concept. Once the species distribution maps had been produced, internal and external control ensured the elimination of obviously wrong records (species outside their natural range). Climate data are based on original climate variables from the Climate Research Unit (CRU) of the University of East Anglia and derived climate variables generated by the ALARM project. Both, CRU and ALARM ensured a high level of internal and external quality control. Data quality for the calculation of the climatic niche characteristics for each butterfly species is high (about 200,000 records for butterfly distribution; well recognised and commonly accepted climate data). Additionally, we provide the number of grid cells which have been used to calculate the climatic species characteristics and the standard deviation to assess uncertainty of the measures.

## Data status and accessibility

### Status

Data set version: v1.3

Latest update: 18.10.2013.

Metadata status: Metadata are complete and stored with the data.

### Accessibility

**Copyright restrictions:** None.

**Proprietary restrictions:** This dataset is freely available for non-commercial scientific use.

**Citation:** Data users must cite this Data Paper properly in any publication that results from an analysis using the provided data as a whole or in parts as: Schweiger O, Harpke A, Wiemers M, Settele J (2013). CLIMBER: Climatic niche characteristics of the butterflies in Europe. ZooKeys 367: 65–84. doi: 10.3897/zookeys.367.6185

**In addition to the Data Paper the resource should be cited as:** Helmholtz Centre for Environmental Research - UFZ (2013). CLIMBER: Climatic niche characteristics

of the butterflies in Europe. 397 records, Online at http://ipt.pensoft.net/ipt/resource.do?r=climber, version 1.3 (released on 3/12/2013), Resource ID: GBIF key: http://www.gbif.org/dataset/e2bcea8c-dfea-475e-a4ae-af282b4ea1c5, Data Paper ID: doi: 10.3897/zookeys.367.6185

#### Collab

**oration:** Data users might consider collaboration and/or co-authorship with the data owners.

**Storage location:** http://ipt.pensoft.net/ipt/resource.do?r=climber

## Data structure

### Dataset file

**File name:** CLIMBER.v.1.3.csv

**Size:** 398 rows, 67 columns; 183 kB.

**Format and storage mode:** ASCII csv, semicolon-delimited; decimal separator: ‘.’.

**Header information:** First row provides variable names.

**Alphanumeric attributes:** Mixed.

**Special characters:** Missing values are indicated by NA.

### Variable definition

Climatic niche characteristics are based on nine climate variables (Table 3). All climate variables represent average values for the period of 1971–2000. Seven statistics are available for each climate variable (Table 4).

**Table 3.** Climatic variables used for the assessment of climatic niche characteristics of the butterflies in Europe.

Name	Definition	Unit	Interpretation
range.size	Distributional range size as number of occupied grids	Grid cells	Sample size
temp	Mean annual temperature	°C	Temperature (STI)
range.ann.temp	Annual range in monthly temperature (warmest month - coldest month)	°C	Continentality
precip	Annual precipitation sum	mm	Precipitation
range.ann.precip	Annual range in monthly precipitation sum (wettest month - driest month)	mm	Oceanity
gdd.feb	Accumulated growing degree days above 5°C from January to February	°C	Temperature corrected for metabolic activity preconditions
gdd.apr	Accumulated growing degree days above 5°C from January to April	°C	Temperature corrected for metabolic activity preconditions
gdd.june	Accumulated growing degree days above 5°C from January to June	°C	Temperature corrected for metabolic activity preconditions
gdd.aug	Accumulated growing degree days above 5°C from January to August	°C	Temperature corrected for metabolic activity preconditions
swc	Soil water content of the upper horizon (0.5 m)	No unit (0-1)	Water availability

**Table 4.** Statistics available for each climate variable describing the niche position and breadth for the butterflies in Europe.

Name	Definition	Interpretation
mean	Mean value of climate variable across the species’ range	‘Optimal’ climatic conditions; niche position
ci.95.low	Lower 95% confidence interval for the mean	Uncertainty of the mean
ci.95.up	Upper 95% confidence interval for the mean	Uncertainty of the mean
min	Minimum value of the climate variable across the species range	Lower climatic limit
max	Maximum value of the climate variable across the species range	Upper climatic limit
sd	Standard deviation of the climate variable across the species range	Niche breadth

We also provide an estimation of species range size (range.size) to assess the number of grid cells used for calculating the climatic species characteristics. For a detailed description of swc see section Climate data. Annual measures are calculated over full 12 month periods, while accumulated growing degree days have been calculated for four periods from January to February, April, June and August to cover a variety of phenological aspects and life cycle stages.

Species range refers to the distributional range according to the 50 km × 50 km CGRS grid cells in which a species was recorded.

### Data anomalies

**Missing values:** NA indicates that a species was only present in one grid cell and thus 95% confidence intervals and standard deviation could not be calculated.

## Appendix

Database of the climatic niche characteristics of the butterflies in Europe (CLIMBER). (doi: 10.3897/zookeys.367.6185.app) File format: Comma-separated values file (csv).
